# Normal Graft Function After Pig-to-Human Kidney Xenotransplant

**DOI:** 10.1001/jamasurg.2023.2774

**Published:** 2023-08-16

**Authors:** Jayme E. Locke, Vineeta Kumar, Douglas Anderson, Paige M. Porrett

**Affiliations:** 1 University of Alabama at Birmingham Heersink School of Medicine, Birmingham

## Abstract

This case series examines the ability of a pig-to-human xenograft to provide life-sustaining kidney function.

Thirty-seven million adults in the US have chronic kidney disease (CKD), many of whom will ultimately progress to end-stage kidney disease (ESKD). Kidney transplant is the gold-standard therapy for patients with ESKD, yet annually, only 25 000 individuals receive a kidney. The gap between supply and demand is so vast that 40% of listed patients die within 5 years while waiting for a kidney transplant. Although xenotransplant represents 1 potential solution for the kidney shortage, previous reports of pig-to-human kidney xenotransplant using a preclinical human brain death model have shown xenograft urine production but not creatinine clearance, a necessary function to sustain life.^1,2^ Thus, no study to date has shown the ability of a xenograft to provide life-sustaining kidney function in a human.

## Methods

In this case series, persons aged 18 years or older declared brain dead whose families provided informed consent for study participation were eligible for study entry after all organ donation options for transplant were exhausted. The decedent received cardiopulmonary support in a critical care setting for the duration of the study. Porcine donor animals with 10 gene modifications, including 4 gene knockdowns and knockouts (*GTKO*, *CMAH*, *B4GALNT2*, *GHR*) and 6 human transgene insertions (*CD46*, *CD55*, *CD47*, *THBD*, *PROCR*, *HMOX1*) as previously described,^1^ were maintained in a pathogen-free facility. General anesthesia was administered for the purposes of porcine kidney procurement, and porcine donors were humanely euthanized thereafter. Ten-gene–edited porcine kidneys were flushed with University of Wisconsin solution, sterile packaged, cold-stored on ice, labeled, and transported via ground to the transplant center. The study was approved by the University of Alabama at Birmingham’s institutional review board (approval No. 300004648) and the institutional animal care and use committee (approval No. 22015). The study followed the Appropriate Use and Reporting of Uncontrolled Case Series in the Medical Literature reporting guideline.

## Results

**Figure 1.**
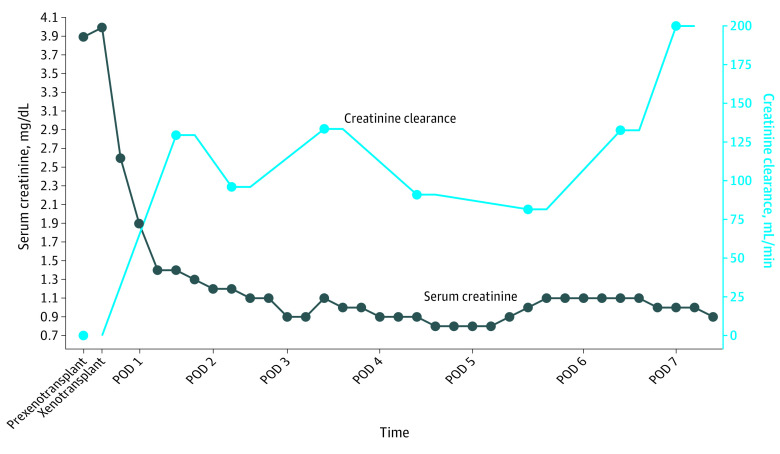
Kidney Function Over Time After 10-Gene–Edited Pig-to-Human Xenotransplant Xenograft-associated declining serum creatinine and increasing creatinine clearance in the absence of native kidneys and dialysis were consistent with life-sustaining kidney function after pig-to-human kidney xenotransplant. POD indicates postoperative day.

**Figure 2.**
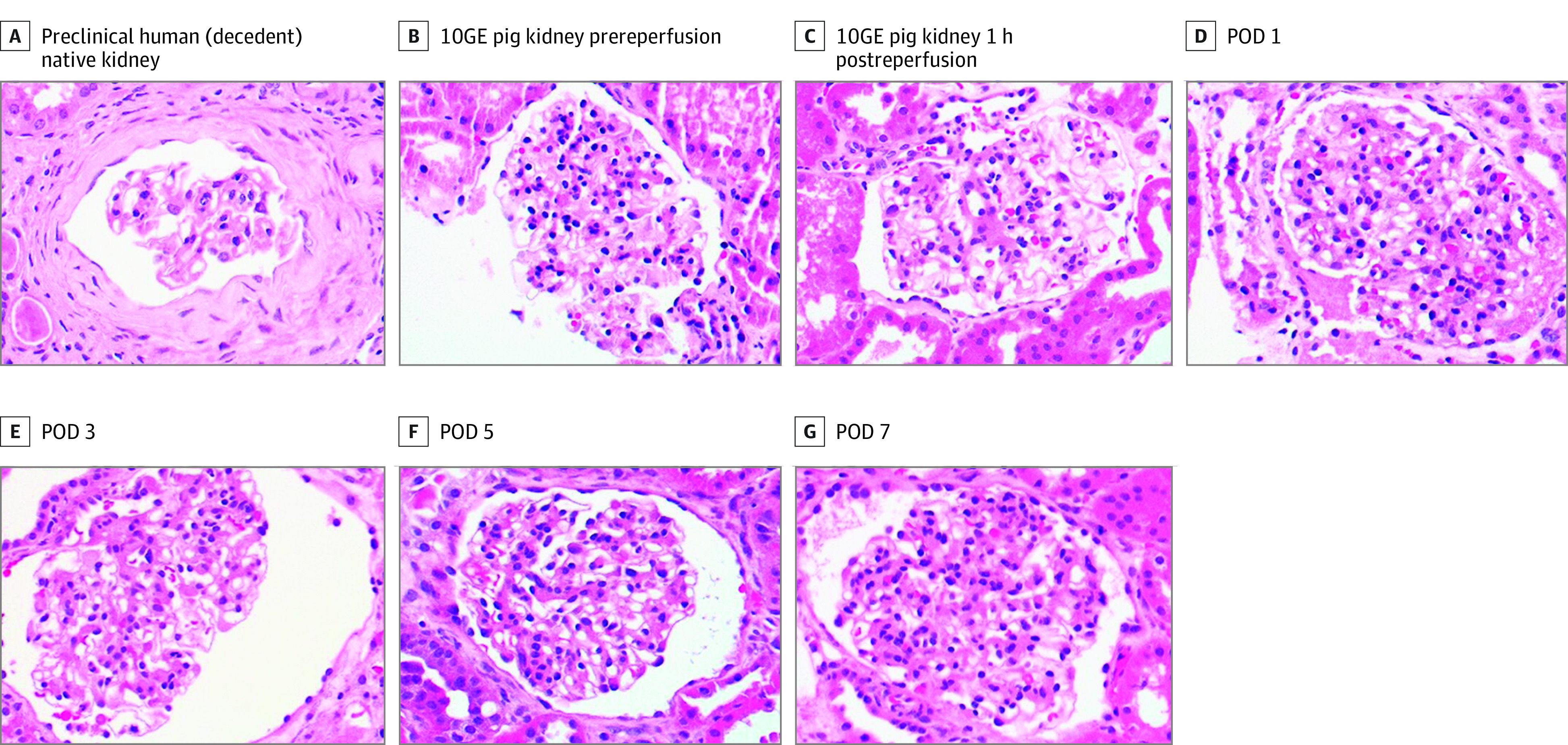
Kidney Histopathology After 10-Gene–Edited (10GE) Pig-to-Human Xenotransplant Original magnification ×40. POD indicates postoperative day.

A male in his 50s who was declared brain dead and had acute kidney injury superimposed on a history of CKD (stage 2) and hypertension underwent bilateral native nephrectomy and cessation of dialysis followed by crossmatch-compatible xenotransplant with 10-gene–edited pig kidneys (UKidney). The decedent received a complement inhibitor (anti-C5; eculizumab) 24 hours before xenotransplant followed by standard induction therapy, including a solumedrol taper, antithymocyte globulin (6 mg/kg total), and rituximab. Maintenance immunosuppression included tacrolimus, mycophenolate mofetil, and prednisone. Goal tacrolimus levels (8-10 ng/dL) were reached by postoperative day (POD) 2 and maintained through study completion. Xenografts were transplanted en bloc with pig vasculature anastomosed to the decedent’s right-side common iliac artery and distal inferior vena cava and the pig ureters anastomosed to the decedent’s bladder. Within 4 minutes of reperfusion, the xenografts made urine, producing more than 37 L in the first 24 hours. Urine concentrated over time, with concurrent decreases in urine volume to a median of 14.1 L (IQR, 13.8-20 L) on PODs 1 to 3 and a median of 5.1 L (IQR, 5-6 L) on PODs 4 to 7. Before xenotransplant, serum creatinine was 3.9 mg/dL after cessation of dialysis and bilateral native nephrectomy. After xenotransplant, serum creatinine decreased to 1.9 mg/dL within the first 24 hours, normalized to 1.1 mg/dL at 48 hours, remained within normal limits through study duration, and was 0.9 mg/dL on POD 7 at study completion. Creatinine clearance also improved (POD 0, 0 mL/min; POD 7, 200 mL/min) (Figure 1). Xenografts were serially biopsied and showed normal histology by light microscopy without evidence of thrombotic microangiopathy (Figure 2).

## Discussion

The findings from this case series show that pig-to-human xenotransplant provided life-sustaining kidney function in a deceased human with CKD. Future research in living human recipients is necessary to determine long-term xenograft kidney function and whether xenografts could serve as a bridge or destination therapy for ESKD. Because our study represents a single case, generalizability of the findings is limited. This study showcases xenotransplant as a viable potential solution to an organ shortage crisis responsible for thousands of preventable deaths annually.

## Notes

Supplement.
Data Sharing Statement
