# Supplementary material for: Normal Graft Function After Pig-to-Human Kidney Xenotransplant
Source: JAMA Surg. 2023 Aug 16;158(10):1106–8. doi: 10.1001/jamasurg.2023.2774 (PMC10433134; doi:10.1001/jamasurg.2023.2774)
Supplement: Supplement. — Data Sharing Statement [file jamasurg-e232774-s001.pdf]

Data Sharing Statement

Locke. Normal Graft Function After Pig-to-Human Kidney Xenotransplant. *JAMA Surg*. Published August 16, 2023. doi:10.1001/jamasurg.2023.2774

Data
Data available: No
